# CONTINUOUS USERS OF LONG-TERM CARE 2.0 IN TAIWAN: PATTERNS AND HEALTH COSTS OVER TIME

**DOI:** 10.1093/geroni/igae098.3162

**Published:** 2024-12-31

**Authors:** Ya-Mei Chen, Shih-Cyuan Wu, Shiau-Fang Chao, Ming-Jen Lin, Chien-Fang Chu

**Affiliations:** National Taiwan University, Taipei City, Taipei, Taiwan (Republic of China); National Taiwan University, Taipei, Taipei, Taiwan (Republic of China); National Taiwan University, Taipei, Taipei, Taiwan (Republic of China); National Taiwan University, Taipei, Taipei, Taiwan (Republic of China); Ministry of Health and Welfare, Taipei City, Taipei, Taiwan (Republic of China)

## Abstract

Taiwan has implemented the Long-Term Care 2.0 (LTC 2.0) Plan since 2017, but only about one third of users continuous to remain in the system after 6 months. The current study examined the patterns of the continuous user and their associations to older adults’ health costs overtime. This study utilized Taiwan’s LTC Plan 2.0 database and the National Health Insurance Plan claim dataset, and included 155,220 clients who continuously utilized any LTC Plan 2.0 services for six months between 2017 and 2019. These users’ health costs were examined in the 12 months after 6 months of HCBS continuous use. Latent class analysis and generalized estimating equations were used to investigate the associations between different service use patterns and the changes in health costs. Five subgroups of LTC use patterns were identified, including home-based personal care (PC) group (n= 99,163, 63.9%), home-based medical care (MC) group (n= 13,304, 8.5%), home-based personal care and medical care (PC/MC) group (n = 9,269, 6.0%), Reablement (RA) group (n= 20,132, 13.0%), and community care (CC) group (n= 13,352, 8.6%). When compared to care recipients in PC/MC group, those in the PC group ($5.84/month, p< 0.001), MC group ($8.21/month, p< 0.001), and CC group ($6.79/month, p< 0.001) had increased more health costs overtime. Such trends are more prominent among those with moderate and severe disability, except those in RA group. The integrated services (PC/MC group) and reablement (RA group) demonstrate slower growth in health costs, particularly among those with moderate and severe disabilities.

